# Anti-neutrophil Cytoplasmic Antibody-Associated Vasculitis Presenting With Disseminated Streptococcus constellatus Pyogenic Infection

**DOI:** 10.7759/cureus.87317

**Published:** 2025-07-05

**Authors:** Erieta Karypidou, Christos Mademlis, Christina Adamichou, Elpida Skouvaklidou, Christos Mavridis, Charalampos Zarras, Polykarpos Flegkas, Elisavet Simoulidou, Aristeidis Kefas, Sofia Chatzimichailidou, Anastasia Sarvani, Theocharis Koufakis, Georgios Damianidis, Athina Pyrpasopoulou

**Affiliations:** 1 2nd Propedeutic Department of Internal Medicine, Hippokration General Hospital Thessaloniki, Thessaloniki, GRC; 2 Department of Rheumatology, Hippokration General Hospital Thessaloniki, Thessaloniki, GRC; 3 4th Department of Internal Medicine, Hippokration General Hospital Thessaloniki, Thessaloniki, GRC; 4 Department of Orthopaedics, Hippokration General Hospital Thessaloniki, Thessaloniki, GRC; 5 Department of Microbiology, Hippokration General Hospital Thessaloniki, Thessaloniki, GRC

**Keywords:** anca-associated vasculitis, infection and autoimmunity, septic polyarthritis, streptococcus anginosus group, streptococcus constellatus

## Abstract

Infections and autoimmunity exhibit a two-way alternate interaction. This report aims to present the case of severe disseminated sepsis leading to the diagnosis of antineutrophil cytoplasmic antibody (ANCA)-positive granulomatosis with polyangiitis. A 49-year-old male presented with fever, anemia, septic polyarthritis, bilateral pulmonary infiltrates with associated acute respiratory distress syndrome, deep vein thrombosis, and acute renal failure. *Streptococcus constellatus* was isolated from blood cultures and joint aspirates. Despite the initiation of targeted treatment, improvement was marginal; respiratory tract involvement worsened, and a parapneumonic fluid collection developed. The patient was investigated for immune system dysregulation. Investigation for hematological abnormalities or neoplasia was negative. An autoimmune antibody profile revealed high positivity for proteinase 3 cytoplasmic-ANCAs. Corticosteroids and rituximab were administered with a good clinical response. Although, in this case, the triggering of autoimmunity through a longstanding infection cannot be ruled out, the persistence of symptomatology despite control of the infection indicates the presence of an underlying autoimmune disease. Patients with ANCA-associated vasculitis are often diagnosed in the context of an infection, which can be encountered especially in the initial period after diagnosis. Accurate differential diagnosis is important to ensure appropriate treatment.

## Introduction

Infections have been etiologically associated with autoimmunity [1,2]. Many different types of infections may lead to, or exacerbate, one or more autoimmune diseases, and a single organism may trigger more than one autoimmune disease. This etiological association is attributed to the development and clonal proliferation of autoreactive T and B lymphocytes [3]. Conversely, infections, frequently involving opportunistic pathogens, are often encountered in patients with a dysregulated immune system [4,5]. Distinguishing between which condition preceded and which is responsible for the presenting symptomatology poses a diagnostic challenge and complicates clinical decision-making and treatment [6]. Lack of, or suboptimal, response to antimicrobial therapy prompts further investigation. We report the case of a previously healthy adult male who presented septic, with disseminated pyogenic infection and, during his hospitalization, was diagnosed with c-antineutrophil cytoplasmic autoantibody (c-ANCA)-positive granulomatosis with polyangiitis (GPA).

## Case presentation

A 49-year-old male was transferred from a regional hospital, following a 72-hour hospitalization, for further investigation and treatment of fever, acute respiratory distress syndrome, acute renal failure, and presumed osteolytic lesions. The patient’s medical history included weight loss, malaise, arthralgias (involving mainly the right shoulder and the right hip/knee joints), and perspiration for the past two months prior to presentation. He had self-administered nonsteroidal anti-inflammatory drugs in large doses. He was a nonsmoker, did not consume alcohol, and his medical records were negative for any chronic medical conditions and/or previous hospitalizations. The patient mentioned attempts to self-extract teeth due to poor oral and dental hygiene before the initiation of symptomatology. At the regional hospital, a computed tomography (CT) scan of the brain, lungs, and abdomen was performed, which showed thrombosis of the left internal jugular vein, bilateral pulmonary infiltrates, osteolytic lesions of the right sternoclavicular joint and right shoulder, and arthritis of the right sacroiliac joint with an adjacent abscess. He had been transfused with three packed red blood cell units due to anemia (with a mildly positive direct Coombs test) and had received two sessions of hemodialysis.

At presentation, the patient had a low-grade fever (37.1°C) and was tachypneic (30 breaths/min), requiring oxygen supplementation of 10 L/min. His chest X-ray showed extensive bilateral infiltrates. The patient had clinical signs of right arm synovitis with restricted movement; his right leg had a similarly decreased range of motion. A right jugular central venous catheter was in place. Upon arrival, his laboratory examinations showed leukocytosis, increased inflammatory markers (C-reactive protein (CRP) and procalcitonin (PCT)), and impaired renal function (Table 1).

**Table 1:** Laboratory parameters at presentation (day 1), during hospitalization (day 7, day 14), and after initiation of immunosuppression. White blood cells (WBC); normal values (nv); hemoglobin (Hb); platelets (PLTs); C-reactive protein (CRP); procalcitonin (PCT); erythrocyte sedimentation rate (ESR)

Lab parameter	Day 1 (admission)	Day 7	Day 14	Day 20 (after three daily courses of 500 mg Solumedrol)	Day 28 (1st dose of rituximab)
WBC (10^3^/μL; nv 4.5-10.5)	26.4	17.0	8.5	13.1	13.1
Hb (g/dL; nv 11.5-14.5)	8.0	7.5	8.5	8.1	7.8
Bilirubin: total/direct (mg/dL; nv 0.3-1.2/ 0.0-0.5)	4.0/2.3	0.75/0.43	1.03/0.45	0.56/0.27	0.53/0.23
PLTs (10^3^/μL; nv 150-400)	276	438		470	
Creatinine (mg/dL; nv 0.8-1.25)	2.92	3.44	1.25	1.05	0.88
CRP (mg/L; nv<6)	264.8	232.0	152.0	65.4	59.1
PCT (mcg/L; nv<0,5)	17	1.67	0.57	0.09	0.66
ESR (mm/1st h; nv<15)	111			89	
24h urine protein (g/24 h; nv<0.15 g)		2.26		4.72	3.15

**Figure 1:**
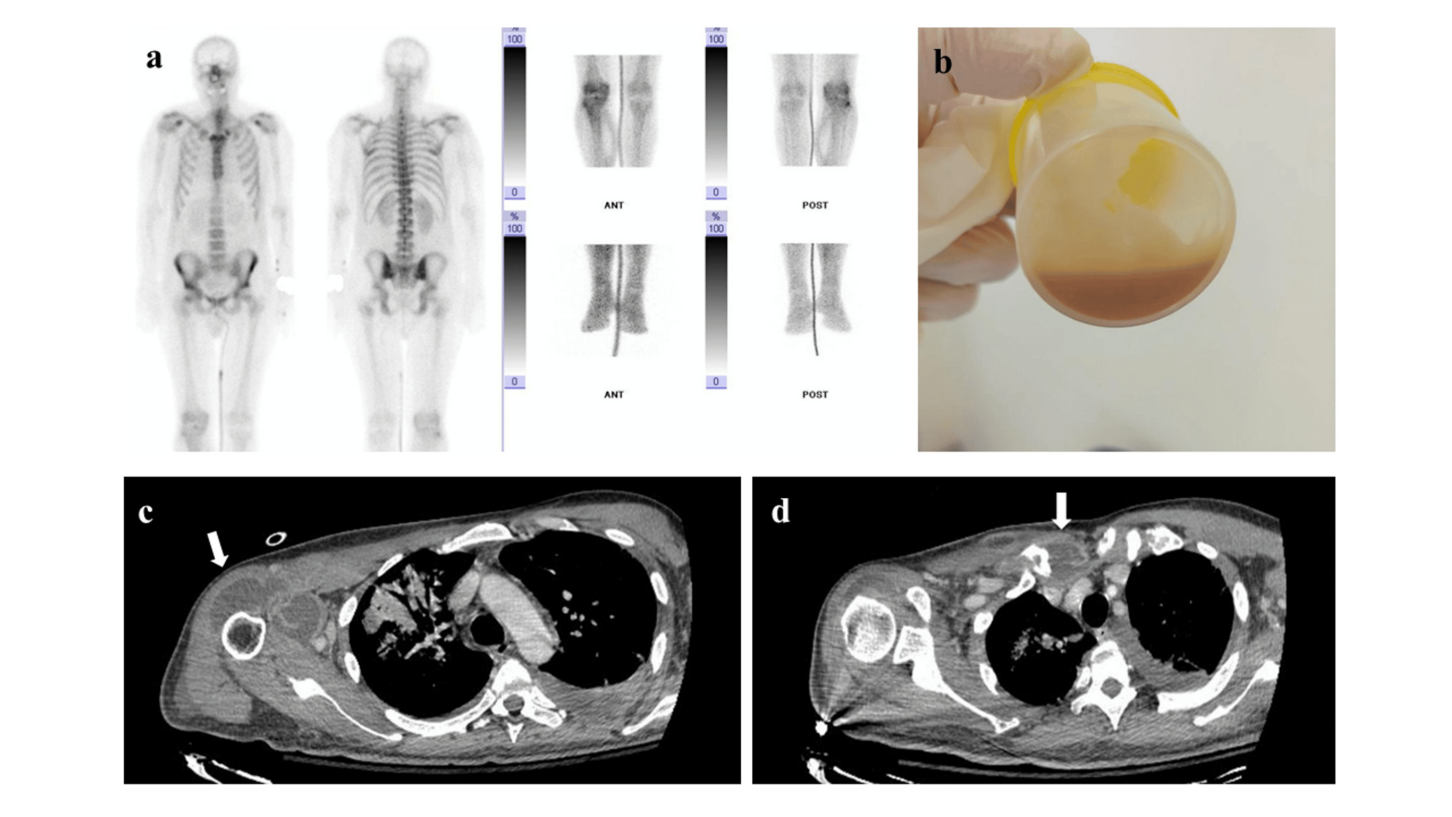
Bone scintigraphy showing polyarthritis involving the sternoclavicular joints, the right shoulder, and the right knee joint (a). Pus aspirated from the right shoulder (b). CT scan showing bursitis of the right shoulder (white arrow) (c). CT scan showing bursitis of the right sternoclavicular joint (white arrow) (d).

Serological screening for acute and chronic hepatitis was negative. Blood and urine cultures were drawn; his antibiotic regimen was modified to piperacillin/tazobactam, linezolid, and doxycycline; and the right shoulder was punctured and lavaged (Figures 1b-1d). Blood cultures and cultures of the arthritic fluid all grew *Streptococcus constellatus*. Piperacillin/tazobactam and doxycycline were discontinued, and ceftriaxone was added to the treatment regimen.

**Figure 2:**
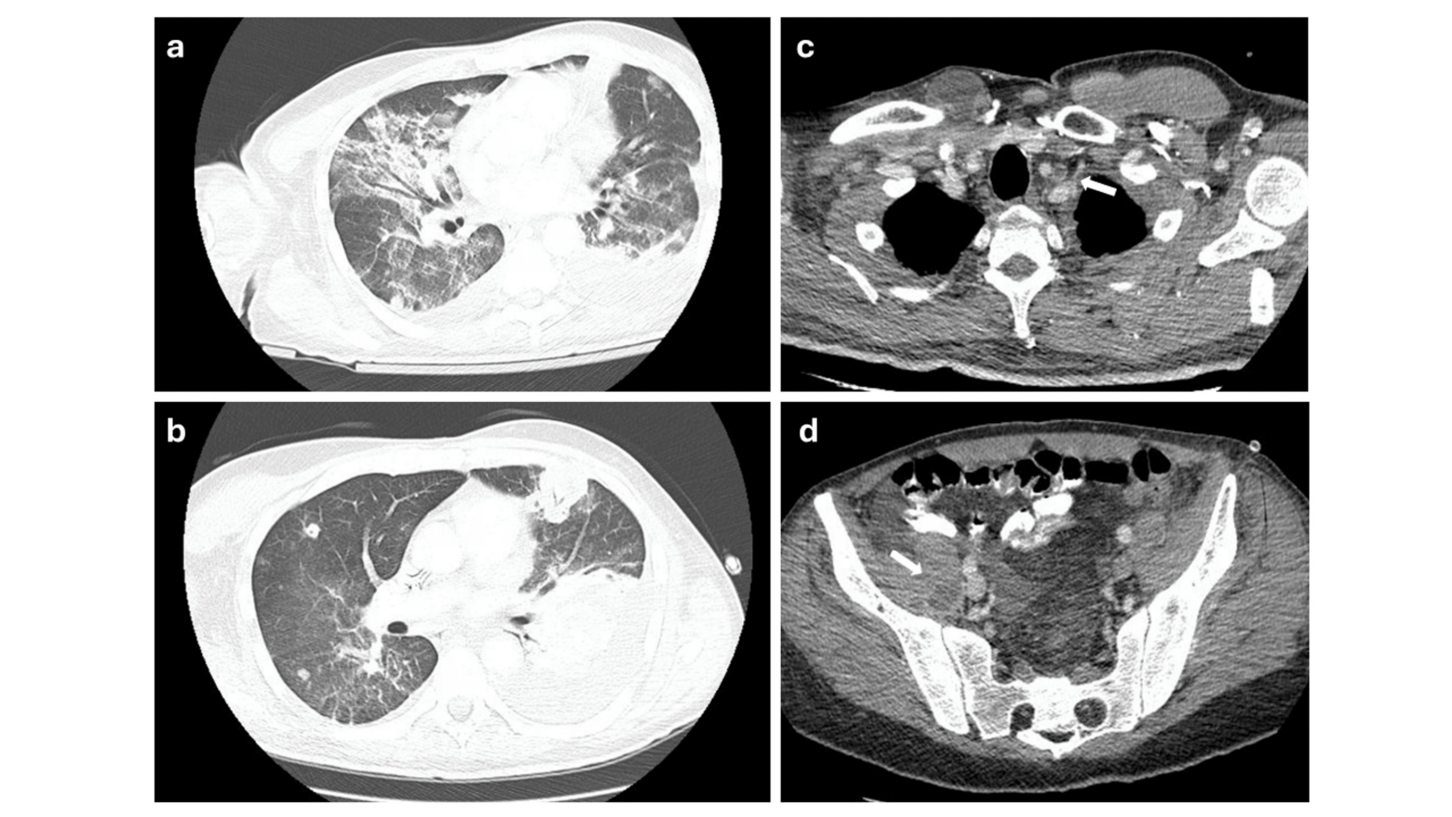
Thorax CT scan at presentation (a) and two weeks later (b). Bilateral pulmonary infiltrates and ground-glass opacities (a). Development of nodular lesions and a left parapneumonic fluid collection (b). Thrombosis of the left internal jugular vein (white arrow) (c). Abscess of the right iliopsoas muscle (white arrow) (d).

A transient clinical improvement was recorded with the implementation of these interventions. However, inflammatory markers (CRP) remained elevated (>150 mg/L) on day 14 (Table 1). Renal function returned to normal levels; however, microscopic hematuria with dysmorphic red blood cells persisted in consecutive urinalyses. Transesophageal echocardiography was negative for valve vegetation. Bone scintigraphy confirmed asymmetrical polyarthritis (Figure 1a) involving, apart from the two previously recognized joints, the right knee joint, and increased tracer uptake of the adjacent muscle/soft tissue. The patient developed left-sided pleural fluid, which was drained, and was identified as an inflammatory parapneumonic fluid collection; culture was negative (Figures 2a-2b).

A screen for autoimmune diseases was requested, which was strongly positive for proteinase 3 (PR3) c-ANCA in repeated samples. Ear, nose, and throat (ENT) consultation revealed nasal septum perforation; a relevant biopsy showed erosions and nonspecific inflammation. Three daily pulses of 500 mg methylprednisolone were administered, and rituximab was initiated upon completion of the antibiotic regimen. The patient improved dramatically, both clinically and in terms of laboratory examinations (day 20, Table 1). Oxygen administration was discontinued, the first dose of rituximab was given, and he was discharged on prophylactic co-trimoxazole and a follow-up appointment in the Rheumatology Outpatient Clinic.

## Discussion

In cases of first-time manifestation and diagnosis of autoimmune diseases in the context of a recent infection, a basic research question is which of the two conditions preceded. Infectious agents have long been known to be one of the principal triggers of autoimmunity, usually by providing an autoantigen mimic, epitope spreading, or bystander activation [7,8]. Conversely, autoimmunity may result from a prolonged, poorly controlled infection, as a consequence of failure of normal immunoregulation to eliminate the pathogen and/or limit the immune response to infection. For example, chronic hepatitis C virus (HCV) infection with persistent antigenic stimulus (viremia) may lead to monoclonal IgM rheumatoid factor production, immune complex formation, and complement activation. HCV infection is found in 70-100% of patients with mixed cryoglobulinemic vasculitis [9]. The induced pathophysiology may or may not, in turn, progress from manifestations of autoimmunity to a full-blown, established autoimmune disease, such as in the case of epigenetic modification of host genes [10]. A characteristic example in the latter case is the causal association of *Porphyromonas gingivalis*, a microbe that is the major causative agent of periodontitis, with citrullination of bacterial and host proteins and induction of rheumatoid arthritis [11].

*S. constellatus* belongs to the *Streptococcus anginosus* group, otherwise known as viridans streptococci. They are commensals of the oral cavity but have repeatedly been implicated in severe infections as community-acquired opportunistic pathogens, usually in patients with underlying comorbidities and/or diseases [12]. Viridans streptococci represent the second most prevalent aerobes isolated from peritonsillar abscesses, next to Group A streptococci. They have additionally been identified as causative pathogens in various pyogenic infections. *S. constellatus* appears to have a propensity for respiratory tract infections but has been isolated from bacteremic patients, patients with gastrointestinal and genitourinary tract infections, and patients with skin and soft tissue diseases; epidemiology in adults may differ from that in children [13,14].

We report the case of a previously healthy 49-year-old male who presented septic and was diagnosed with bacteremia and disseminated pyogenic abscesses caused by *S. constellatus*, and subsequently with c-ANCA GPA. To our knowledge, this is the first case of autoimmunity potentially causally associated with this pathogen. In contrast to Group A streptococcus, which is known for its immunogenic potential, Group B streptococci have rarely been associated with the induction of autoimmunity [15,16]. Patients with GPA often present with concurrent infections at the time of diagnosis [17]. In general, infections, especially involving the respiratory tract, are more common in these patients in the first year of diagnosis. Given the severity of the patient’s clinical presentation and disseminated infection, the administered treatment of choice, after infection control with targeted antibiotics, was pulse steroids and rituximab, to which the patient responded well [18].

## Conclusions

This case presents a typical clinical dilemma of infection-driven autoimmunity on the one hand and a case of severe infection in a patient with immune dysregulation on the other hand. Key clinical and laboratory features in favor of an underlying infection-related autoimmune flare include failure to achieve remission despite appropriate management targeting the responsible pathogen. Detection of autoantibodies with concomitant persistence of a systemic inflammatory response syndrome and/or systemic symptomatology (fever, fatigue) with organ involvement is a key clue that indicates the need for further investigation and supports optimal clinical decision-making.
